# Neuroimmune Interaction in the Regulation of Peripheral Opioid-Mediated Analgesia in Inflammation

**DOI:** 10.3389/fimmu.2016.00293

**Published:** 2016-08-02

**Authors:** Susan Hua

**Affiliations:** ^1^School of Biomedical Sciences and Pharmacy, University of Newcastle, Callaghan, NSW, Australia; ^2^Hunter Medical Research Institute, New Lambton Heights, NSW, Australia

**Keywords:** peripheral nervous system, immune cells, neuroimmune, inflammation, pain, opioids

## Abstract

Peripheral immune cell-mediated analgesia in inflammation is an important endogenous mechanism of pain control. Opioid receptors localized on peripheral sensory nerve terminals are activated by endogenous opioid peptides released from immune cells to produce significant analgesia. Following transendothelial migration of opioid-containing leukocytes into peripheral sites of inflammation, opioid peptides are released into a harsh milieu associated with an increase in temperature, low pH, and high proteolytic activity. Together, this microenvironment has been suggested to increase the activity of opioid peptide metabolism. Therefore, the proximity of immune cells and nerve fibers may be essential to produce adequate analgesic effects. Close associations between opioid-containing immune cells and peripheral nerve terminals have been observed. However, it is not yet determined whether these immune cells actually form synaptic-like contacts with peripheral sensory terminals and/or whether they secrete opioids in a paracrine manner. This review will provide novel insight into the peripheral mechanisms of immune-derived analgesia in inflammation, in particular, the importance of direct interactions between immune cells and the peripheral nervous system.

## Introduction

Peripheral opioid mechanisms of endogenous pain control are potent and of clinical relevance. In addition to animal studies, a sizeable body of clinical literature has clearly shown that opioid receptors localized on peripheral sensory nerve terminals can be activated by both exogenous opioid agonists, as well as by endogenous opioid peptides expressed in immune cells, to produce significant analgesia (1–8). This local opioid-mediated analgesia is devoid of the central side effects of systemic opioid administration (e.g., respiratory depression, sedation, and nausea) and has a relative lack of tolerance after repeated administration of peripheral opioid agonists in inflamed tissue (1, 9, 10). In the early stage of inflammation, granulocytes (esp. neutrophils) are the major opioid-containing leukocyte, whereas at later stages of inflammation, monocytes/macrophages and lymphocytes (esp. activated T- and B-cells) predominate (11–14). Inflammation increases the expression of opioid peptides as well as their mRNA transcripts encoding their precursor proteins within these immune cells (14, 15), with β-endorphin (β-END) from pro-opiomelanocortin (POMC) being the most prominent (7, 16, 17). Studies to date suggest that only a finite number of the total immune cell population actually produce opioid peptides and home to lymph nodes. This is supported by the observation that β-END and POMC mRNA were less abundant in circulating lymphocytes than in those in lymph nodes (14, 18). Ongoing research is focused on differentiating this sub-population of opioid-producing leukocytes for the design of novel targeted therapies. Of even greater interest is what happens once the immune cells enter the inflamed tissue, especially the interaction between the immune cells and peripheral sensory nerve fibers. The proximity of immune cells and nerve fibers may be essential in inflammation, as the overall increased metabolic environment within inflamed tissue increases the activity of opioid peptide metabolism (19–22). As a result, successful pain control may rely on the immune system being even more selective about the location at which opioid peptides are released for efficient and effective pain control (21, 23, 24). This review will provide novel insight into the peripheral mechanisms of immune-derived analgesia in inflammation, in particular, the importance of direct interactions between immune cells and the peripheral nervous system.

## Mechanisms of Peripheral Opioid-Mediated Analgesia

**Figure 1:**
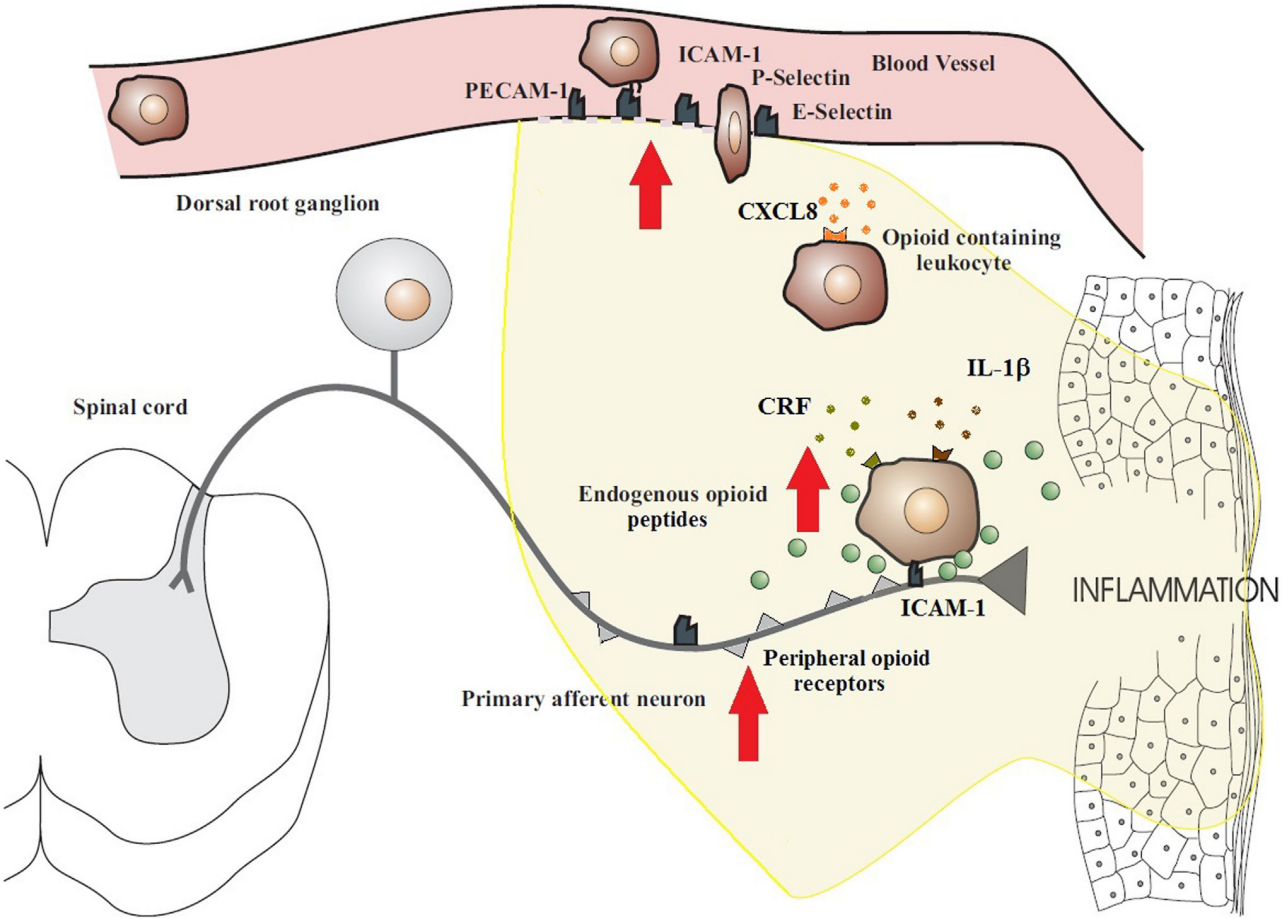
**Migration of opioid-containing immune cells and opioid release within inflamed tissue**. Adhesion molecules interact with their respective ligands to facilitate endothelial transmigration of immune cells. In response to stress or releasing agents (e.g., CRF, IL-1, and CXCL8), the immune cells secrete opioid peptides. Opioid peptides or exogenous opioids bind to opioid receptors on primary afferent neurons, leading to analgesia. The immune cells, depleted of opioids, then migrate to regional lymph nodes. The arrows denote an increased expression within inflamed tissue of cell adhesion molecules, opioid receptors, endogenous opioid peptides, and receptors for ligands that trigger opioid release on the surface of immune cells (e.g., CXCR2, IL-1 receptors, and CRF receptors). All these enhance the analgesic activity of the peripheral opioid pathway in inflammatory conditions. Figure adapted from Ref. (2).

With the duration of inflammation, the number of infiltrating immune cells as well as total opioid peptide content increases steadily at the site of tissue injury. Leukocyte homing, in general, is a multistep process involving the sequential activation of various adhesion molecules located on immune cells and on the vascular endothelium (1, 2) (Figure 1). Initially, circulating leukocytes tether and roll along the vascular endothelial cell wall, a process mediated by selectins on leukocytes (L-selectin) and endothelial cells (P- and E-selectin) (6, 25). Leukocytes are then activated by chemokines released from inflammatory cells and presented on the luminal surface of the endothelium (26, 27). This subsequently leads to upregulation and increased avidity of leukocyte integrins, in particular CD49d/CD29 and CD18, which mediate the firm adhesion of leukocytes to endothelial cells by interacting with members of the immunoglobulin superfamily (e.g., ICAM-1) (26, 27). Thereafter, the cells transmigrate through the endothelium directed predominantly by PECAM-1 expressed on endothelial cells at intercellular junctions and are then directed to the sites of inflammation. All these molecules are constitutively expressed and are upregulated in inflammation, except L-selectin, which is rapidly shed upon activation (26, 28). The relatively low expression of L-selectin on opioid-containing leukocytes is most likely due to its shedding required for leukocyte extravasation (26, 28).

**Figure 2:**
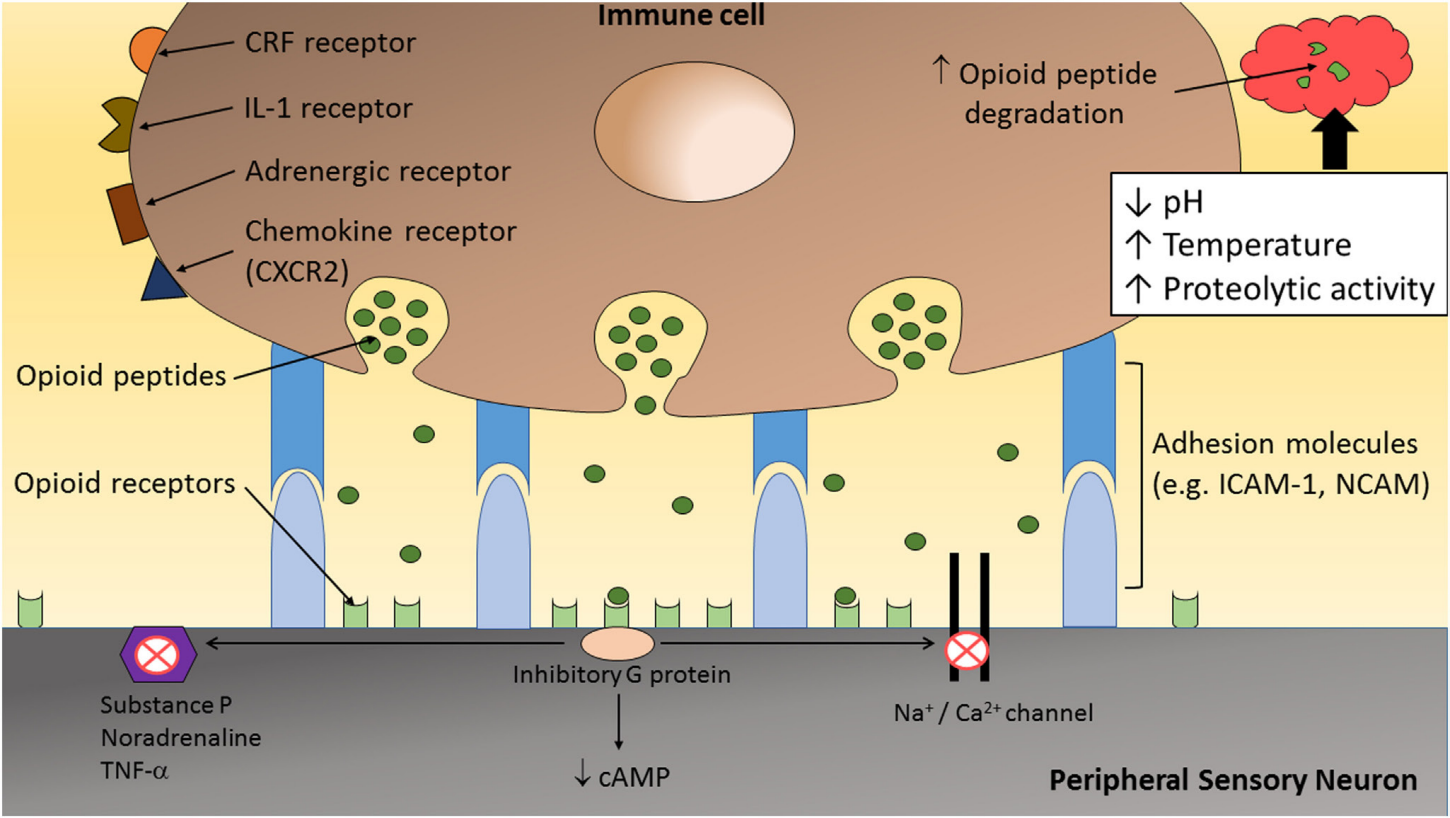
**The inflammatory milieu is associated with an increase in temperature, low pH, and high proteolytic activity, which together has been suggested to increase the degradation of opioid peptides (1, 23)**. Therefore, direct adhesion between opioid-containing immune cells and peripheral sensory neurons, *via* adhesion molecules (e.g., ICAM-1 and/or NCAM), may be necessary to release opioid peptides within the effective range of peripheral opioid receptors to produce adequate analgesia. On activation by opioid agonists, opioid receptors undergo conformational changes allowing intracellular coupling of inhibitory G proteins to the C terminus of opioid receptors. This leads to inhibition of calcium and/or sodium channels and a decrease in the level of neuronal cAMP. In addition, opioids reduce the excitability of nociceptors, the propagation of action potentials, and the release of excitatory and/or pro-inflammatory factors (e.g., substance P, TNF-α, and NA) from peripheral sensory neurons.

In peripheral inflammatory states, opioid-containing immune cells “home” to the inflamed tissue where they release opioid peptides and then travel to local lymph nodes (1, 16). In particular, CD4+ T cells are able to recirculate from the blood, through tissues, into the lymphatic system, and back to the blood. These immune cells exit from the vascular compartment *via* specialized high endothelial venules (HEV) in lymphoid organs. Lymphocyte traffic across the HEV may increase substantially within 3 h following an immune response and by as much as 10-fold over the first 48 h of the response (29, 30). Multiple endogenous factors are able to trigger the release of opioid peptides from immune cells, including environmental stimuli [e.g., stress-induced release of sympathetic neuron-derived noradrenaline (NA)] (31) and local inflammatory factors [e.g., corticotropin-releasing factor (CRF), interleukin-1β (IL-1β), and chemokine CXCL8 (also known as IL-8)] (Figure 1) (2, 18, 21). It has been observed that inflammation upregulates the production of endogenous CRF, IL-1β, and CXCL8 in inflamed tissue as well as the expression of their respective receptors on leukocytes (32). In addition, adrenergic α1, β2, and to a lesser degree, α2 receptors are expressed on β-END-containing inflammatory cells located in close proximity to sympathetic nerve fibers in inflamed paws (31). It should be noted that these factors can also affect other cells at the site of tissue injury to further promote inflammation, thus contributing to the dynamic pro-inflammatory and anti-inflammatory balance. Opioid release from immune cells has been demonstrated to be calcium dependent, which is consistent with a regulated pathway of release from secretory vesicles, similar to neurons and endocrine cells (16, 33). Subsequently, the opioid peptides penetrate the damaged perineurial sheath and activate opioid receptors on peripheral terminals of sensory neurons to produce endogenous analgesia (6, 8) (Figure 2). Increasing studies have also suggested an anti-inflammatory role for peripheral opioids (4, 34). Several mechanisms have been postulated, including inhibition of NA, substance P, and TNF-α release from neuronal cells (35, 36). The function of NA in inflammation is contested with evidence being provided for both a positive role (37) and a negative role (38).

## Inflammation Enhances Degradation of Opioid Peptides

The precise interaction occurring between opioid-containing immune cells and peripheral sensory neurons is only beginning to be elucidated. Inflamed tissues have been shown to have increased metabolic breakdown rates for opioid peptides (19–22). Hence, it is likely that, for adequate analgesia to occur, direct interaction between these cells are required to allow the release of opioid peptides in close proximity to peripheral opioid receptors on sensory neurons (Figure 2). Following tissue injury, the extracellular matrix comprises a myriad of inflammatory mediators (e.g., hydrogen ions, cytokines, and chemokines) and enzymes (39, 40). Activated leukocytes may modify the composition of the extracellular matrix by secreting cytokines and degradative enzymes such as matrix metalloproteinases (MMPs), heparanases, and serine proteases (40). Therefore, following the transendothelial migration of opioid-containing leukocytes into peripheral sites of inflammation, opioid peptides are released into a harsh milieu associated with an increase in temperature (21, 39, 41), low pH (21, 39, 41, 42), and high proteolytic activity (19, 20, 22, 40, 43). Together, this microenvironment has been suggested to increase β-END degradation, which is supported by the short-lasting antinociceptive effect following a single local injection of an exogenous opioid peptide (21).

Endogenous opioid peptides are rapidly degraded by human peripheral blood proteases, giving a half-life of approximately 5 min for enkephalins and 40 min for β-END (19, 22, 24). However, within peripheral inflamed tissue, opioid peptides are exposed to hydrolysis by additional groups of enzymes, including plasma soluble peptidases, peptidases present in the membrane of immune cells, and peptidases released by immune cells (19). In addition, peptidases bound to the extracellular surface of neurons (44) and those associated with opioid receptors (45) degrade opioids in their microenvironment. Consequently, pro-enkephalin (PENK)-derived peptides are very susceptible to proteolytic action resulting in short-lasting central and peripheral antinociceptive actions (19, 46). Endothelial cell enzymes have also been shown to degrade human β-END into various peptide fragments (43). Administration of inhibitors of enzymatic degradation of these peptides, including enkephalinase and amino-peptidase inhibitors, has been shown to augment the duration of action of opioid peptides (46).

Furthermore, tissue acidosis may be responsible for the enhanced activity of various enzymes and the breakdown of substances, including denaturation of proteins and peptides (47). In fact, average proton concentrations as acidic as pH 5.5 have been observed in inflamed tissues, although this may, however, underestimate the true degree of tissue acidification in the inflammatory foci (39). Several inflammatory processes are responsible for this localized acidosis, including the release of various chemical mediators of pain such as hydrogen ions and the local production of lactic acid (39, 42). In addition, activated neutrophils have been suggested to generate large amount of metabolic acids (e.g., succinic, butyric, propionic, hydrobromic, and hypochlorous acid) (40, 42, 48) and to release an estimated 150 mmol H^+^ ions/liter cells (42). These immune cells further enhance the extrusion of acid and thus contribute to tissue acidosis through the activation of various H^+^ transport processes, in order to maintain their cytosolic pH within physiological limits (42).

Taken together, the analgesic effects of endogenous opioid peptides may depend considerably on their site of secretion from immune cells. It is suspected that the “clouds” of endogenous opioid peptides released from immune cells within inflamed tissues are rapidly surrounded and hydrolyzed by peptidases, resulting in negligible peripheral antinociception (1). Therefore, the release of opioid peptides from immune cells in close apposition to peripheral sensory neurons would maximize the potential for analgesic effects (1, 23) (Figure 2).

## Interaction Between Immune Cells and Neurons in Peripheral Analgesia

Increasing evidence indicates that the nervous and immune systems are not disparate entities. Immune cells have been shown to interact directly with neurons, with comparisons having been made in the literature between neuronal and immunological synapses (49, 50). Previous studies have reported the innervation of lymphoid organs (51–53), skin (54, 55), eye (56), respiratory tract (57–59), gastrointestinal tract (60–64), liver (65), and the CNS (66, 67) by nerve terminals directly adhering or in close proximity to leukocytes. In line with these findings, close association between peripheral nerves and opioid-containing immune cells have previously been observed (1, 23, 68). *In vitro* studies have demonstrated consistent alliance between lymphocytes containing opioids and cultured DRG nerves (23), while *in vivo* studies have observed this same phenomenon in peripheral inflamed tissues with primary afferent nerves (68). It is plausible that this firm adherence between immune cells and primary afferent nerve fibers may have a functional role in releasing opioid peptides close to opioid receptors within inflamed tissue to provide adequate analgesia.

### Anatomical and Functional Neuroimmune Interactions

Anatomical and functional relationships between nerve fibers and immune cells have been highlighted in the literature (49, 50, 69–72). Reports have described the non-random spatial association and bidirectional communication of nerves and immune cells in a variety of tissues in which actual membrane–membrane contacts have been observed (51–53, 57, 58, 60, 65). This concept of a dialog between the immune and sensory nervous system has been based on three observations. First, nerve terminals have been found in direct contact with immune cells (50, 71, 73). Anatomical studies have reported origin, pattern of distribution, and targets of nerve fiber populations supplying lymphoid organs (51–53, 74, 75). For example, electron microscopy has revealed direct contact between noradrenergic nerve terminals and lymphocytes in the spleen (53, 74, 76). The nerve endings were observed forming long smooth zones of contact with lymphocyte plasma membranes, creating a small cleft of only 6 nm (50, 76). It should be noted that gap junctions are generally 2 nm and classical synapses are 20 nm in width (50). In addition, many of the terminals were found to indent into a lymphocyte, and the apposing membranes were often prominent or slightly thickened (76). These contacts have been suggested to promote bidirectional and chemically mediated transmission between nerves and immune cells with transmitter release and postsynaptic receptor activation (52, 53, 74, 75). In particular, cytokines and opioids released by macrophages and lymphocytes can alter neural NA release from presynaptic varicosities (53, 76). Thus, the likelihood of both local paracrine secretion of NA into the splenic parenchyma and true neuroimmune synapses with lymphocytes, exists, which may represent a key link between the autonomic and immune system.

Second, both organ systems share common receptors and ligands (50, 71, 73). In several studies, significant concentrations of binding sites for a number of neurotransmitters and peptides have been identified on the surface of immune cells and neurons. These receptors have been shown to respond *in vivo* and/or *in vitro* to the neural substances, and their manipulation can alter immune responses (71, 72, 75). This direct influence of the nervous system on cellular immune response is evident in the liver given that lymphocytes and other immune cells expressing opioid receptors were regularly found in close apposition to nerve fibers containing dynorphin (a high affinity endogenous kappa opioid receptor ligand) in the liver of both mice and rats (65). This neuroimmune interaction was suggested to have functional roles in inflammation.

Finally, mutual ligand–receptor interactions lead to significant changes in cellular functions in both systems (21, 39, 41). For example, interactions between nerves and inflammatory cells have been shown to lead to a number of important physiological responses in the lungs (57–59, 65, 77). Studies have suggested that contact between eosinophils and cholinergic nerves may be responsible for vagal hyperreactivity by triggering eosinophil degranulation and a subsequent increase in acetylcholine release from the nerves (57, 58). This direct adherence was dependent on the interaction between the eosinophil integrins CD11/18 and VLA-4 with the neuronal adhesion molecules ICAM-1 and VCAM-1 (57, 58). These cell adhesion molecules are expressed in response to cytokines present in the inflammatory milieu, with inhibition of either adhesion molecule preventing eosinophil-nerve binding (57, 58).

This intimate association of immune cells and neurons in various tissues has been proposed as one of the anatomical bases of communication between the immune and the nervous systems. However, the specificity of anatomical associations between these interactions is beginning to be understood. These synaptic-like contacts may provide the transmitter, or specifically opioid peptides, in even higher concentration for a more immediate effect than is available at a distance (49, 50, 70, 71). Therefore, a functional role following direct interaction between opioid-containing immune cells and peripheral sensory neurons may be possible (23) with the enhanced recruitment of lymphocytes, the upregulation of opioid peptides, opioid receptors, and cell adhesion molecules in inflammatory conditions (1, 6).

### Establishing Functional Connectivity between Neuronal and Immune Cells

Close associations between opioid-containing immune cells and peripheral nerve terminals have been observed (23, 68). However, it is not yet determined whether these immune cells actually form synaptic-like contacts with peripheral sensory terminals and/or whether they secrete opioids in a paracrine manner. In order to substantiate productive interactions at a cellular level between peripheral nerves and the immune system, evidence needs to be accumulated that the criteria established for synaptic connectivity are met (49, 50, 68, 70, 78).

A synapse is a stable adhesive junction between two cells across which information is relayed by directed secretion. Specific qualities of a synapse, irrespective of the cells involved, have previously been described (78). Synapses are utilized in both the nervous and immune systems to directly convey and transduce highly controlled secretory signals between their constituent cell populations. The neuroimmune synapse refers to specialized zones between neurons and immune cells or antigen-presenting cells (APC), and, therefore, can be thought of as a hybrid structure between neuronal and immunological synapses (49, 70, 78). Reports in the literature have clearly established that the immune and nervous systems share common mediators (50, 71, 73). Not only can cells in both systems synthesize and release these mediators but also they both can show physiological responses based on the presence of specific receptors (49, 70, 78). For example, immune cell function within the spleen has often been the focus of neuroimmune research because this secondary lymphoid organ is densely innervated by the sympathetic nervous system. In particular, at the electron microscopic level, it has been shown that noradrenergic nerve terminals form intimate contact with the surface membrane of T-lymphocytes and APCs of the peri-arteriolar lymphoid sheath of the spleen, with thickening of presynaptic cellular membranes and concentration of vesicles containing neurotransmitters at neuron–immune cell junctions (52, 76). This neuroimmune junction meets the criteria for synaptically-mediated neurotransmission, including local bidirectionality through cytokines and neurotransmitters from immune cells that modulate the release of sympathetic neurotransmitters from nerve terminals (53, 74, 76).

Cell–cell interactions *via* adhesion molecules are important in the maintenance of communication between cells (49, 78). Evidence already exists for direct cell adhesion between neurons and immune cells involving cell adhesion molecules [e.g., ICAM-1 (23, 54, 58, 79), ICAM-5 (67), VCAM (54, 58), selectins (66), and NCAM (23)]. Although little is known of the consequences of this interaction, it is, however, expected to be relevant in inflammation (54, 58) and neuronal damage (67, 79). The nervous system and immune system utilize these specialized cell surface contacts to directly convey and transduce highly controlled secretory signals between their constituent cell populations. The synaptic structure comprises central active zones of exocytosis and endocytosis encircled by adhesion domains (80). Surface molecules that may be incorporated into and around the active zones contribute to modulation of the functional state of the synapse (49). The potential roles of adhesion molecules at synapses include stability, target recognition, and synaptic differentiation (81, 82). However, the mechanisms that localize molecules to specific subdomains remain unclear. Therefore, the identity of the cell adhesion molecules on the apposed membranes and their local concentration may be important determinants on synapse numbers and their location (81, 82).

The release of chemical mediators into the inflammatory milieu has been reported to increase the expression of various adhesion molecules (6, 26, 28). In particular, NCAM and ICAM may be important in mediating adequate analgesia in inflammatory pain by facilitating firm adhesion between opioid-containing immune cells and peripheral sensory neurons (23) (Figure 2). For example, intraplantar injection of the monoclonal antibody for NCAM, prior to the induction of inflammation, significantly reduced the antinociceptive response (paw pressure and paw thermal thresholds) produced by CRF or cold water swim stress in a dose-dependent manner (23). Anti-NCAM-treated rats responded normally to intraplantar fentanyl. In addition, β-END-containing immune cells within treated and untreated rats were histologically verified to have similar densities, suggesting no effect on leukocyte extravasation into inflamed tissue. *In vitro* studies showed a significant reduction in the number of lymphocytes adherence to DRG neuronal cultures following anti-NCAM and anti-ICAM-1 treatment compared to untreated cultures, thus supporting the notion that opioid-containing immune cells must adhere to peripheral sensory neurons to provide effective analgesia (23).

## Modulation of Immune Cell Adherence by Opioids

Peripheral inflammatory pain can be effectively controlled by an interaction of opioids released by immune cells in close proximity to opioid receptors on peripheral sensory nerve terminals. Although direct contact between primary cultured DRG neurons and lymphocytes have been observed (23), whether this interaction is of functional relevance in peripheral inflammation is not yet established. This adhesion may also be partly mediated by opioid receptors, as shown by the effects of β-END on adhesion between cultured DRG neurons and lymphocytes (23). Exogenous application of β-END significantly attenuated lymphocyte adherence to nerve fibers compared to control, and this was completely and significantly reversed with naloxone. This may highlight an additional anti-inflammatory role for opioids in peripheral analgesia. Immune cell-derived opioids released locally may interfere with this direct neuroimmune interaction, resulting in dissociation and possibly migration of immune cells back to regional lymph nodes (1, 23). However, if direct cell adhesion itself does not elicit opioid release from immune cells, then it is expected that agents such as CRF will trigger such release.

There is growing evidence that opioid peptides are potent modulators of cellular immune response, which can enhance or inhibit immune functions (83–89). Opioids including β-END (85, 86, 90, 91), met-enkephalin (85, 86, 91), and morphine (84) have been shown to modulate the adherence of immune cells to the endothelium. In particular, β-END and met-enkephalin, at physiological concentrations (10^−8^ and 10^−6^ M), enhanced the adherence and migration of human monocytes and neutrophils across capillary endothelial cells into inflamed tissues (85, 87). However, at higher concentrations of β-END (10^−3^ M) and met-enkephalin (10^−5^ M), chemotaxis of these immune cells into inflammatory sites decreased (85). The adherence of immune cell to the endothelium was suggested to involve opioid modulation of the expression of adhesion molecules, with quantitative studies confirming an increased number of integrin (CD11b and CD18) receptors on neutrophils at lower opioid concentrations (85). Furthermore, morphine has been demonstrated to attenuate leukocyte rolling and adhesion in both arterioles and venules *via* stimulation of nitric oxide production, which, in turn, downregulates the expression of adhesion molecules (e.g., selectins and integrins) on endothelial cells (84). It is, therefore, likely that immune cell-derived opioids may attenuate the adherence of lymphocytes to DRG neurons following release within peripheral inflamed tissue (23).

## Conclusion

Increasing evidence exists for a functional role in neuroimmune interactions between opioid-containing immune cells and peripheral sensory neurons within inflamed tissue. Since a fundamental goal is to understand synapse assembly at the molecular level, techniques such as electron microscopy, electrophysiology, and immunocytochemistry are powerful methods for characterizing structural, functional, and molecular attributes, respectively (92). This will provide novel insight into the peripheral mechanisms of immune-derived analgesia in inflammation, and the potential development of new therapeutic strategies utilizing this alternative analgesic pathway to counteract peripheral inflammatory pain.

## Author Contributions

SH was responsible for drafting and revising the article.

## Conflict of Interest Statement

The research was conducted in the absence of any commercial or financial relationships that could be construed as a potential conflict of interest.
